# Well-Defined Polyethylene Glycol Microscale Hydrogel Blocks Containing Gold Nanorods for Dual Photothermal and Chemotherapeutic Therapy

**DOI:** 10.3390/pharmaceutics14030551

**Published:** 2022-02-28

**Authors:** Ben Newland, Johannes Starke, Chiara Bastiancich, Diana P. N. Gonçalves, Laura J. Bray, Wenxin Wang, Carsten Werner

**Affiliations:** 1 Leibniz-Institut für Polymerforschung Dresden e.V., Max Bergmann Center of Biomaterials Dresden, Hohe Str. 6, 01069 Dresden, Germany; johannes.starke@web.de (J.S.); laura.bray@qut.edu.au (L.J.B.); werner@ipfdd.de (C.W.); 2 School of Pharmacy and Pharmaceutical Sciences, Cardiff University, Cardiff CF10 3NB, UK; 3 Aix-Marseille Univ, CNRS, INP, Inst Neurophysiopathol, 13344 Marseille, France; chiara.bastiancich@univ-amu.fr; 4 Department of Chemistry and Biochemistry, Kent State University, Kent, OH 44242, USA; dgoncal1@kent.edu; 5 Charles Institute of Dermatology, School of Medicine, University College Dublin, Belfield, D04 V1W8 Dublin, Ireland; wenxin.wang@ucd.ie; 6 Center for Regenerative Therapies Dresden and Cluster of Excellence Physics of Life, Technische Universität Dresden, 01062 Dresden, Germany

**Keywords:** glioblastoma, photodynamic therapy, microscale hydrogels, polyethylene glycol, gold nanorods

## Abstract

Local drug delivery offers a means of achieving a high concentration of therapeutic agents directly at the tumor site, whilst minimizing systemic toxicity. For heterogenous cancers such as glioblastoma, multimodal therapeutic approaches hold promise for better efficacy. Herein, we aimed to create a well-defined and reproducible drug delivery system that also incorporates gold nanorods for photothermal therapy. Solvent-assisted micromolding was used to create uniform sacrificial templates in which microscale hydrogels were formed with and without gold nanorods throughout their structure. The microscale hydrogels could be loaded with doxorubicin, releasing it over a period of one week, causing toxicity to glioma cells. Since these microscale hydrogels were designed for direct intratumoral injection, therefore bypassing the blood–brain barrier, the highly potent breast cancer therapeutic doxorubicin was repurposed for use in this study. By contrast, the unloaded hydrogels were well tolerated, without decreasing cell viability. Irradiation with near-infrared light caused heating of the hydrogels, showing that if concentrated at an injection site, these hydrogels maybe able to cause anticancer activity through two separate mechanisms.

## 1. Introduction

A broad range of biomaterials are being developed for local delivery of therapeutics specifically to the required site in the body [1]. For brain cancers such as high-grade gliomas, in particular glioblastoma, whose prognosis can be very poor, locally administered therapeutics may offer improved outcomes over the current treatment strategy [2]. The potential advantages of using drug delivery systems to release the drug at the tumor site (or in the tumor resection cavity following cytoreductive resection) include raising the local dose of the drug at the tumor site and repurposing drugs that cannot pass the blood–brain barrier. Local drug delivery therefore gives the opportunity to use drugs such as doxorubicin (which exhibits greater cytotoxicity to glioblastoma cells than the currently clinically used temozolomide) for combinatory approaches aiming at bypassing cancer cells chemoresistance [3]. Approximately 65–75% of glioblastoma patients undergo surgical resection of the tumor [4], which leaves a cavity into which drug delivery systems can be placed [5]. However, for non-resectable tumors, injectable drug delivery systems with adapted physicochemical and mechanical properties could also be administered intratumorally.

Hydrogels that form in situ offer the possibility to be injected intratumorally or into the tumor resection cavity [6,7]. In situ-forming hydrogels can be used to deliver drugs loaded into nanocarriers such as micelles [8] or nanoparticles [9]. This approach is attractive, as the hydrogel will take the form of the irregularly shaped cavity and can adhere to its borders [5]. However, in principle, if biomaterials can be created that are small enough to be injected, they can be synthesized, pre-formed, and loaded with therapeutics, prior to injection, without the need for in situ gelation. Our group previously created such systems, using microscale cryogels [10] or small fragments of hydrogels [11] for focal delivery of doxorubicin to orthotopic breast cancer xenografts. However, in both cases, control over the delivery system size was poor, leading to some cryogels or hydrogel fragments being larger than others, necessitating a means of producing highly reproducible and standardized systems with appropriate dimensions for the intended application.

Sacrificial template-assisted synthesis strategies offer a route to creating uniform biomaterials with well-defined dimensions determined by the template shape [12,13,14,15]. Although microcontact printing is commonly used to print inks such as proteins and other molecules onto a surface [16,17], it can also be used to perform solvent-assisted micromolding to create uniform polystyrene sacrificial templates [18,19]. Furthermore, microscale precision can be obtained when patterning hydrogels from these templates [20]. We therefore hypothesized that a template-based strategy could be used to create well-defined hydrogels with high reproducibility.

Photopolymerization of polyethylene glycol (PEG)-based acrylate monomers has been previously undertaken within the resection cavity to encapsulate drug-loaded delivery systems [8,9]. However, in this study we aimed to create a PEG-based hydrogel that could be loaded directly with the drug doxorubicin and release it in a controlled fashion without the need for an additional carrier.

Photothermal therapy has recently been shown to be a promising therapeutic approach in pre-clinical GBM studies [21]. Since it causes tumor destruction using heat rather than chemical therapeutics, it holds potential to overcome the limitations of standard therapeutics caused by glioblastoma heterogeneity and radioresistance/chemoresistance. For this reason, in this work, we also aimed to investigate whether gold nanorods, which heat up upon irradiation by near-infrared light [22], could be incorporated into the hydrogels for a combined photothermal and chemotherapeutic effect.

In this study, we synthetized and characterized PEG-based hydrogels. Then, we tested the biocompatibility of the free scaffolds and the cytotoxicity of the drug-loaded scaffolds towards C6 cells. Finally, we included gold nanorods in the system and tested it for photothermal ablation using the same cell line.

## 2. Materials and Methods

### 2.1. Materials and Reagents

All reagents were purchased from Merck (Darmstadt, Germany) unless otherwise stated. All water used in the experiments was first subjected to MilliQ purification.

### 2.2. Synthesis of a Template Featuring Microscale, Rectangular Cavities

Polydimethylsiloxane (PDMS) stamps were fabricated by casting PDMS on silicon masters which were designed with symmetric arrays of square-shaped cavities with width of 50 µm or 100 µm, using photolithographic etching (GeSiM, Großerkmannsdorf, Germany). The etched surface of the masters measured 1 cm × 1 cm, and the cavities were 10 µm deep. PDMS (Sylgard 184 silicone elastomer kit, Dow Corning, Wiesbaden, Germany) was cast in a microcontact printer with a 10 mm^2^ stamp body (GeSiM, Großerkmannsdorf, Germany). The PDMS stamp was cured at 100 °C for 2 h before removal from the silicon master. Using a microcontact printer (µCP 3.0, GeSiM) in a semi-automated fashion, the PDMS stamp in the body was dipped into ethyl acetate before being pressed onto a 0.4 mm-thick polystyrene sheet (Evergreen Scale Models, Des Plaines, IL USA). After 15 min, the stamp was retracted, leaving behind a polystyrene mold of the silicon master. Immediately prior to the synthesis of the block microgels, the polystyrene molds were plasma-cleaned (air/vacuum, PDC-002, Harrick, Ithaca, NY, USA) to increase the hydrophilicity of the mold surface for greater wetting.

### 2.3. Synthesis of Microscale Hydrogels

**Figure 1:**
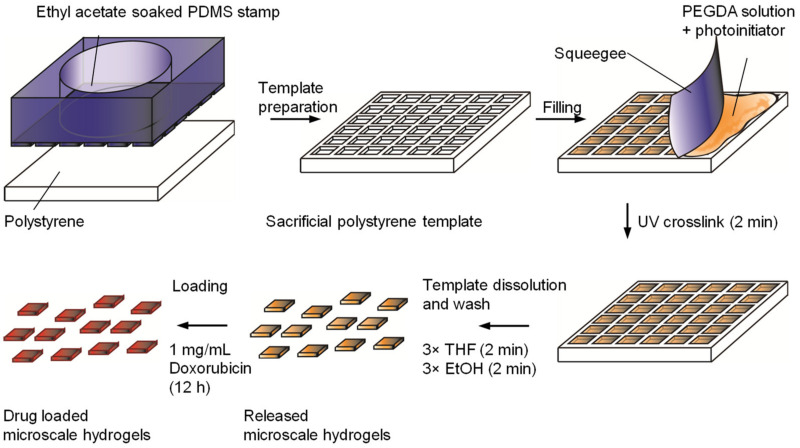
A schematic depiction of the process of microscale hydrogel formation and loading with doxorubicin, repurposed for use in local glioblastoma therapeutics. Utilization of semi-automated solvent-assisted microcontact molding of sacrificial templates allows the production of large numbers of hydrogels with precise dimensions, without batch-to-batch variation.

Polyethylene glycol diacrylate (PEGDA) with a molecular weight of 575 Da was diluted in phosphate-buffered saline (PBS) to a concentration of 0.5 g/mL. Hydroxy-2-methylpropiophenone was used as a photoinitiator and added to the PEGDA solution at a final concentration of 0.01% *w*/*v*. Two microliters of this PEGDA precursor solution was added to the plasma-treated templates, using a polystyrene sheet to remove the excess, as shown in Figure 1.

The filled template was placed under nitrogen flow for 2 min before being exposed to UV light (8000 mW/cm^2^, Delolux 04, Delo GmBH, Windach, Germany) for 2 min for photopolymerization to occur.

Hydrogels containing gold nanorods were synthesized by adding previously prepared nanorods [22] into the PEGDA precursor solution at a final concentration of 120 µg/mL prior to photopolymerization as above. The nanorods were imaged by transmission electron microscopy (TEM) using a Zeiss Libra 200 TEM with an accelerating voltage of 200 kV [23].

The block-shaped hydrogels, with depths of 10 µm and widths of either 50 or 100 µm, were removed from the template by adding the template to 1 mL of tetrahydrofuran (THF). Gentle shaking and 1 min of water bath sonication released the hydrogels, as the template began to dissolve. These were then removed and washed three times with 1 mL THF, centrifuging at 13,400 rpm for 10 min to pellet the hydrogels allowing supernatant removal. The hydrogels were subject to another two washes in ethanol before drying to obtain a dry weight followed by resuspension in the appropriate medium.

### 2.4. Empty and Filled Template Characterization

The topography and dimensions of the empty templates and the hydrogel filled polystyrene templates (air-dried) were analyzed by both multi-pinhole confocal microscopy and scanning electron microscopy (SEM). Multi-pinhole confocal microscopy was performed using a μsurf explorer (NanoFocus AG, Oberhausen, Germany) with a 10× objective (Olympus, Hamburg, Germany). We generate 3D height maps, and recorded profile sections using μSoft analysis software (version 7) (NanoFocus AG) as reported previously [24]. SEM analysis was carried out using a XL30 ESEM-FEG microscope (Philips, Amsterdam, The Netherlands) in high-vacuum mode using an accelerating voltage of 10 kV. The samples were first fixed to aluminum stubs with carbon-conductive tape, then sputter-coated with gold via an SCD 050 sputter coater (BAL-TEC, Pfäffikon, Switzerland) for 40 s at 40 mA.

### 2.5. Rheological Analysis of Macroscale Hydrogels

Using the same precursor solution as above, disks of PEGDA hydrogels were created in a mold 8 mm in diameter and 1 mm in depth. These were placed in PBS for 6 h prior to rheological measurement. An ARES LN2 rotational rheometer (TA Instruments, Eschborn, Germany) was used to measure the storage modulus (G’) by oscillatory shear experiments. The distance between the oscillatory plates was reduced to 90% of the original gel height, and then frequency sweeps were performed from 1 to 100 rad/s, at 25 °C with a strain amplitude of 2%. Four replicates were performed, and an average was calculated.

### 2.6. Loading the Hydrogels with Doxorubicin and Characterizing the Release

We added 960 µg of hydrogels (either 50 or 100 µm width) to 50 µL of a doxorubicin (LC Laboratories, Woburn, MA, USA) stock solution (1.5 mg/mL in water containing 0.1% *v*/*v* dimethyl sulfoxide (DMSO)) and left them for 24 h at room temperature. Unloaded doxorubicin was removed by careful aspiration of the supernatant and by washing once in 2 mL PBS.

To characterize the release of doxorubicin from the hydrogels, they were transferred to a 0.4 µm-pore filter insert for a 24-well plate and filled with 2 mL of PBS per well. At 4 h and then 1, 2, 3, 6 and 7 days, PBS was removed and stored (−80 °C) and replaced with fresh PBS. Then, 100 µL of the samples was analyzed by absorbance at 482 nm (Spark^®^ plate reader (Tecan Trading AG, Männedorf, Switzerland) in comparison to a standard curve as reported previously [25]. Four replicates were undertaken, and a mean value was used.

The doxorubicin-loaded hydrogels were also visualized via a fluorescence microscope set up for time-lapse imaging (Zeiss, Oberkochen, Germany; 10× lens). Images were acquired every hour for 48 h and analyzed using 4.8 AxioVision (Zeiss) with ImageJ (NIH) software (version 1.58j8) to determine the mean fluorescence intensity and create composite (brightfield + 488 nm) images.

### 2.7. Glioma Cell Culture and Viability Analysis

C6 glioma cells were purchased from Merck (92090409-1VL) and cultured in Dulbecco’s Modified Eagle Medium/F12 mix (Catalog number: 11320033, Gibco/ThermoFisher, Dreieich, Germany) supplemented with 10% *v*/*v* fetal bovine serum (FBS, Biochrom, Cambridge, UK), penicillin (100 U/mL), and streptomycin (100 μg/mL). The cells were maintained using standard sterile cell culture conditions and incubated at 37 °C with 5% carbon dioxide. Then, 5000 C6 glioma cells were seeded per well in a 96-well plate and incubated for 24 h prior to experimentation. We prepared 960 µg of 50 or 100 µm-width hydrogels, either loaded with doxorubicin or without drug, in 2 mL of cell culture medium. A serial dilution of the hydrogels was then prepared, from 480 µg/mL to 30 µg/mL, in cell culture medium, and 100 µL of each concentration was added per well to the C6 cells. After 24 or 48 h, the cells were imaged using an inverted light microscope (Olympus IX73 (Shinjuku, Japan)), and cell viability was measured via the PrestoBlue metabolic activity assay, following the manufacturer’s protocol, in comparison to untreated control cells as reported previously [23]. These experiments were carried out with four replicates, and a mean value was calculated.

### 2.8. Photothermal Studies Using Gold Nanorods-Containing Hydrogels with or without Loaded Doxorubicin

Two experiments were carried out to characterize the temperature change of 50 µm hydrogels containing nanorods. The first involved irradiation of the hydrogels whilst they were still in the template, performed at 21 °C, the second utilized hydrogels in 100 µL of PBS at a concentration of 480 µg/mL and was performed in an incubator at 37 °C. An 800 nm laser (Roithner Lasertechnik GmbH, Vienna, Austria) was used 10 cm from the sample for 10 min (5 mW/mm^2^). The temperature was monitored using a FLIR E6 infrared camera (FLIR Systems GmbH, Frankfurt am Main, Germany) and recorded every 5 s (minimum interval) to every minute (maximum interval). To analyze the cytotoxicity caused by photothermal ablation, C6 glioma cells were cultured and seeded into 96-well plates as described above. Then, 100 µL of cell culture medium was added, containing nanorod-filled hydrogels (either with or without prior loading of doxorubicin (as described above)) at a concentration of 480 µg/mL. After 1 h in the incubator, each sample well was irradiated (whilst at 37 °C) for 10 min, then left for the remaining 23 h. PrestoBlue was used to analyze the viability of the cells in comparison to the cell-only control. As an additional control, cells without hydrogels were irradiated for 10 min. The experiments were repeated four times, and a mean value was calculated.

### 2.9. Statistical Analysis

For all cell culture experiments, four replicates were carried out, and the mean was value plotted with error bars representing +/− standard deviation. The cell-free system used for photothermal analysis was examined with three replicates for the control hydrogels and four for the nanorod filled hydrogels. Statistical analysis was performed for the photothermal cytotoxicity experiment using Graph Pad Prism software (version 6.01), using a one-way ANOVA and Tukey’s post-hoc analysis; asterisks mark statistically significant differences with respect to the control, indicated as ** *p* < 0.01, *** *p* < 0.001, and ns = no statistical significance.

## 3. Results

Polystyrene templates with block-shaped micro-structured cavities were produced by solvent-assisted microcontact molding using PDMS stamps that were created from two different silicon masters. The template was filled with a PEGDA precursor solution, which was photo-polymerized within the template as outlined in Figure 1. The photoinitiator hydroxy-2-methylpropiophenone was utilized for ease of mixing with relatively hydrophilic monomer solutions and lack of cytotoxicity [26].

**Figure 2:**
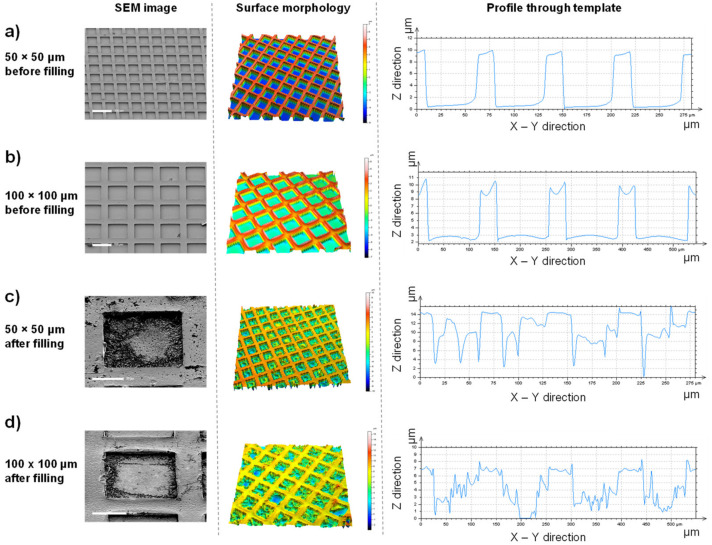
Well-defined microscale templates can easily be filled with a hydrogel precursor solution. Scanning electron microscope (SEM) images (left panel) with corresponding multi-pinhole confocal microscopy height maps (middle panel) and cross-sectional data (right panel) show unfilled templates of 50 µm (**a**) and 100 µm (**b**) widths. Analysis of the filling of these templates was performed after hydrogel formation but before template removal, for both 50 µm- (**c**) and 100 µm- (**d**) width templates. Scale bars represent 100 µm (**a**,**b**), 20 µm (**c**), and 50 µm (**d**).

The empty and filled templates were characterized by scanning electron microscopy and multi-pinhole confocal microscopy (Figure 2a,b). These templates measured 10 mm^2^ and had cavities 10 µm deep, that were either 50 or 100 µm square, with depth and profile measurements showing faithful replication of the template cavity dimensions. Despite being analyzed in a dehydrated state, filling of the template could still be observed by both characterization methods (Figure 2c,d).

**Figure 3:**
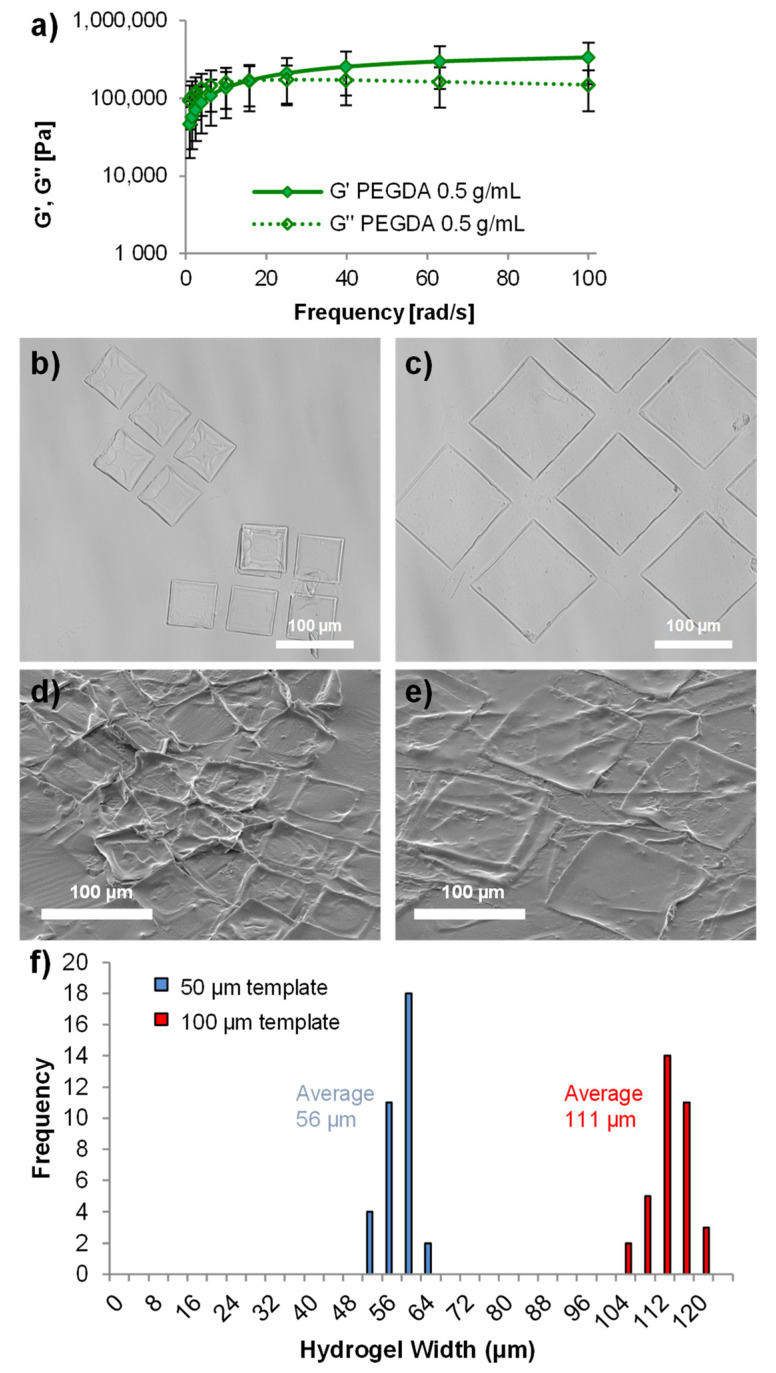
Characterization of the microscale hydrogels. Rheological measurements of bulk hydrogels formed from the relatively high monomer concentration used (0.5 g/mL), showing the flexibility of the hydrogels formed (**a**) (*n* = 4, error bars represent ± standard deviation). Light microscope images of 50 µm hydrogels (**b**) and 100 µm hydrogels (**c**) whilst hydrated in PBS. Scanning electron microscope images of 50 µm hydrogels (**d**) and 100 µm hydrogels (**e**) whilst dehydrated under vacuum, showing that the overall shape of the material was maintained. Characterization of the size of the microscale hydrogels in PBS shows (**f**) that they had a tight size distribution and swelled to a size slightly larger than the template they were formed within. All scale bars represent 100 µm.

Whilst technically a hydrogel, the PEGDA monomer was used at a concentration of 0.5 g/mL, giving rise to microscale blocks with a higher solid content than we previously used with brain tissue (0.07 g/mL) [27]. The higher solid content (and hence higher crosslinking density) resulted in hydrogels with a high Young’s modulus as determined by rheometry (Figure 3a). So, although these hydrogels were not stiffness-matched to the brain tissue (with a stiffness typically less than 5 kPa [28,29]), they were not rigid like wafers developed for implantation into brain cancer resection cavities [30,31]. Hydrogels for drug delivery to glioblastoma are considered an alternative route to local drug delivery that maybe well suited to applications in a resection cavity [5]. The mechanical robustness of these microscale hydrogels allows them to be removed from the template and easily pipetted using standard laboratory equipment without breaking (Figure 3b–e). Analysis of the hydrogel sizes indicated that they swelled by approximately 112% of their original template size once removed from the template (Figure 3f).

Local drug delivery as a strategy for therapeutic delivery to brain cancers would allow the administration of drugs that cannot pass the blood–brain barrier [32]. Local delivery therefore vastly increases the range of potential chemotherapeutic drugs that can be utilized. For example, researchers have shown that doxorubicin, a mainstay therapeutic for breast cancer, has greater potency than temozolomide against glioblastoma cells [3].

**Figure 4:**
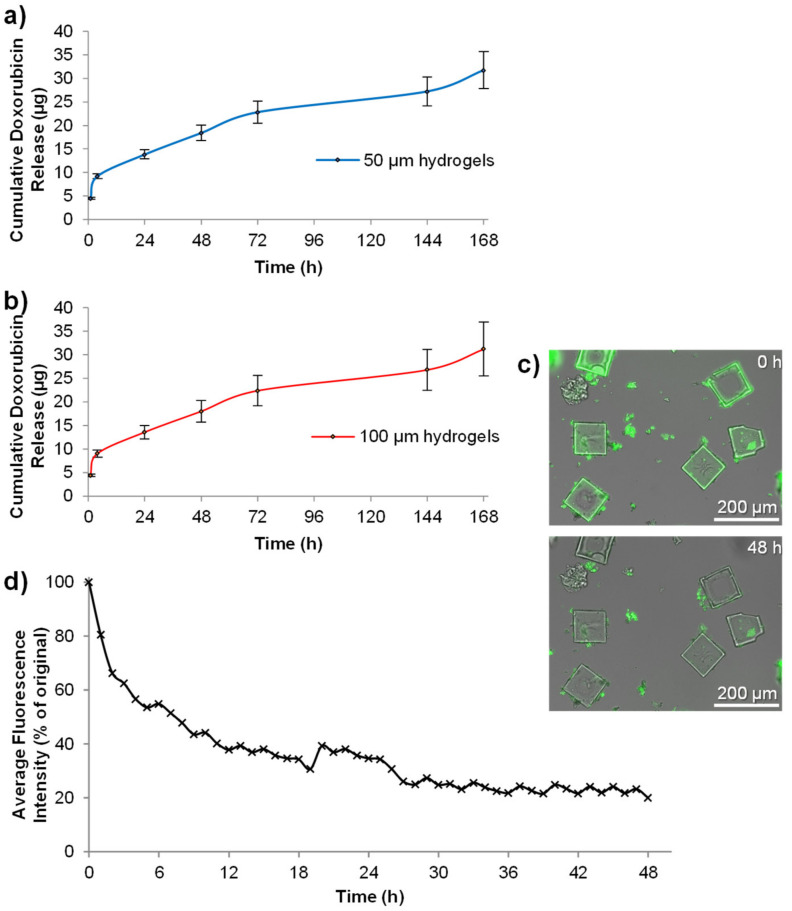
Doxorubicin is released from the microscale hydrogels steadily over 7 days. We show that 50 µm width hydrogels (**a**) and 100 µm width hydrogels (**b**) released doxorubicin into PBS over a period of 7 days (*n* = 4, error bars represent ± standard deviation). Microscale hydrogels could also be visualized by time-lapse fluorescence microscopy due to the intrinsic fluorescence of doxorubicin (**c**), showing a drop in fluorescence intensity over 48 h (**d**).

In silico modelling of an affinity-based drug delivery system composed of polyethylene glycol (PEG) and heparin showed that doxorubicin has a little electrostatic affinity for PEG [10]. Controlled delivery from PEG-based hydrogels that do not contain negatively charged moieties therefore requires a high solid content, with a small mesh size, to control the diffusion of doxorubicin through the polymer network. Indeed, the hydrogels prepared herein released doxorubicin for 7 days (longest time tested) (Figure 4).

Both 50 µm and 100 µm hydrogels showed very similar release characteristics (Figure 4a,b). This was likely due to fact that both hydrogel sizes were formed from the same components at the same concentration and that the same mass, not number, of hydrogels were used for both studies. Furthermore, whilst the widths of the hydrogels were different, they both had matching depths of 10 µm. We were concerned that the “sin” conditions created by full exchange of the medium at each time point might affect the release profile (i.e., more frequent changes in the first 24 h might stimulate faster release). However, time-lapse fluorescence microscopy analysis of 100 µm hydrogels (Figure 4c), without medium changes, confirmed that doxorubicin release was indeed slightly faster over the first 5 h than the remaining 43 h (Figure 4d). However, these hydrogels did not exhibit the burst release profile that some delivery systems exhibit when loaded with doxorubicin [15,33,34,35].

**Figure 5:**
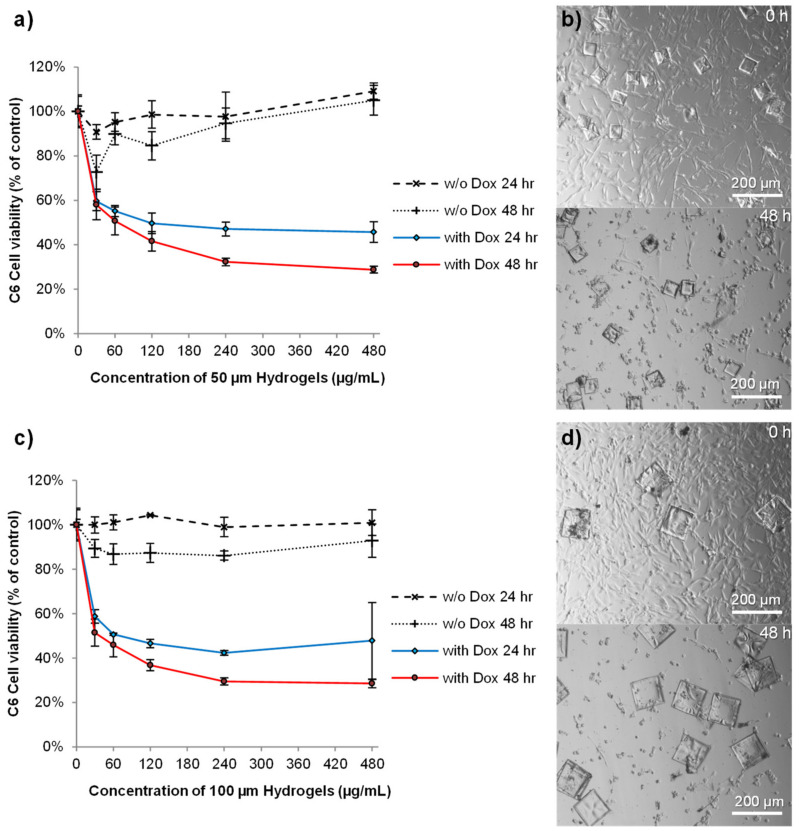
Doxorubicin-loaded microscale hydrogels reduce glioma cell viability, whilst unloaded hydrogels are not cytotoxic. PrestoBlue^®^ analysis of C6 glioma cell viability for 50 µm-width hydrogels (**a**) and 100 µm-width hydrogels (**c**) showed that unloaded hydrogels were not toxic at any concentration analyzed, yet, reduced cell viability in a dose-dependent manner when loaded with doxorubicin (*n* = 4, error bars represent ± standard deviation). Light microscope images of 50 µm-width hydrogels (**b**) and 100 µm-width hydrogels (**d**) loaded with doxorubicin, immediately after addition of the hydrogels at a concentration of 480 µg/mL and then again after 48 h. Scale bars represent 200 µm.

The C6 glioma cell line was used to analyze the cytotoxicity profile of the hydrogels, either unloaded or loaded with doxorubicin and/or gold nanorods for photothermal ablation. C6 cells are a relatively fair representation of many glioblastoma characteristics and have been extensively used for testing therapeutics [36]. Unloaded hydrogels (with both 50 and 100 µm widths) did not cause a reduction in cell viability at the maximum concentration tested (480 µg/mL) (Figure 5). This data are in agreement with a previous study using the same monomer and photoinitiator to create PEG nanotubes that showed no toxicity at all concentrations tested [15]. Furthermore, polyethylene glycol dimethacrylate hydrogel rods were shown to be well tolerated in rodent brain for extended time periods [37]. In contrast, doxorubicin-loaded hydrogels reduced cell viability in a dose-dependent manner (Figure 5a,c). The effect was rapid, with a hydrogel concentration of 480 µg/mL causing a 50% loss in cell viability over 24 h, as indicated by large areas devoid of cells by 48 h (Figure 5b,d). In agreement with the release studies, similar effects of the doxorubicin-loaded hydrogels were observed, regardless of the hydrogel width.

**Figure 6:**
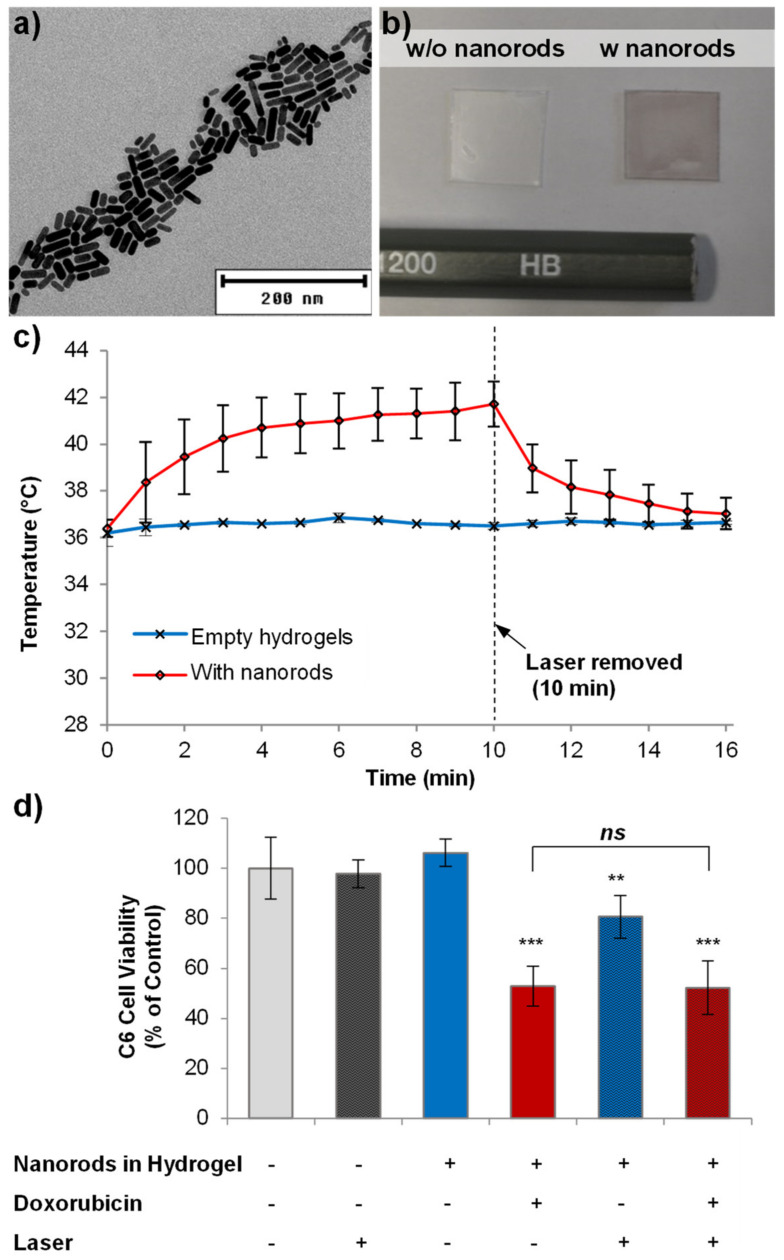
Microscale hydrogels can be loaded with gold nanorods for combined drug delivery and photothermal ablation. Transmission electron microscope image of gold nanorods (**a**), with comparison images of unloaded and nanorod-loaded microscale hydrogels whilst still in their 100 µm template (**b**) showing the slight darkening of the hydrogels. Irradiation with near-infrared light (800 nm) increased the temperature of a 480 µg/mL solution of nanorod-loaded hydrogels to 41.7 °C (**c**) (*n* = 3, error bars represent standard deviation). When administered directly to C6 glioma cells, nanorod-loaded hydrogels caused a slight decrease in cell viability when irradiated for 10 min (**d**), though a combination of photothermal ablation and doxorubicin delivery did not result in greater cell loss than doxorubicin delivery alone (*n* = 4, error bars represent ± standard deviation, asterisks represent ** *p* < 0.01, *** *p* < 0.001 and ns = no statistical significance).

Gold nanorods have been developed for photothermal ablation of glioblastoma, via selective nanorod cell uptake [22,38]. By tuning the nanorod shape characteristics, gold nanorods can absorb near-infrared light and generate heat [39]. Since only a moderate rise in temperature can reduce the viability of C6 tumors [40], we wanted to analyze whether photothermal ablation via gold nanorod-loaded hydrogels could be achieved either alone or in combination with doxorubicin release. The incorporation of gold nanorods, 28 ± 7 nm in length and 9 ± 2 nm in width [22] (Figure 6a), into the hydrogels caused a slight darkening of the hydrogels (Figure 6b), though no other differences in properties were observed. We found that 100 µm-width, nanorod-loaded hydrogels showed a rapid rise in temperature upon irradiation with near-infrared light whilst still in the template, reaching a maximum temperature of 50 °C (Supplementary Information Figure S1). However, when suspended in 100 µL of cell culture medium at a concentration of 480 µg/mL, a rise in temperature of the medium to 41 °C could be observed after 10 min of irradiation (Figure 6c).

During cytotoxicity analysis, 10 min of irradiation caused no reduction in the viability of control cells without hydrogels (Figure 6d). The addition of hydrogels (100 µm width) at a concentration of 480 µg/mL, but in the absence of the laser, also caused no reduction in viability. However, combining near-infrared light with the nanorod-filled hydrogels resulted in a small but statistically significant reduction in viability (81% viability). However, when doxorubicin-loaded, nanorod-filled hydrogels were used, in the absence of the laser, cell viability was reduced to 53% due to doxorubicin cytotoxicity. Unfortunately, no additional effect of combining the doxorubicin-loaded hydrogels with the laser was observed, as the viability remained similar (52% viability). Therefore, no combinatorial effect of dual photothermal ablation and doxorubicin release was achieved.

Together, these data indicate that doxorubicin played the key role in cell death. In vivo hyperthermia studies with C6 cells indicate that reaching temperatures over 43 °C for one hour [40] would achieve greater therapeutic efficacy. Whilst the hydrogel alone study showed these hydrogels could surpass these temperatures (Figure S1 from Supplementary Materials), filling the hydrogels with more nanorods and lengthening the duration of irradiation should improve the heating effect of the surrounding medium. It is also worthy to note that tuning the nanorod dimensions for a larger aspect ratio could result in a greater heating ability.

Whilst photothermal therapy is more commonly applied to other cancer types, translation to neuro-oncology would require positioning of the fiberoptic cable transmitting the near-infrared light either into the resection cavity or, in the case of non-resectable tumors, through a catheter, directly into the tumor. Such experimental strategies could be considered for cancers such as glioblastoma, which still have a poor prognosis.

The development of photothermal therapies for brain tumors such as glioblastoma is hampered by the fact that photothermal agents must concomitantly possess appropriate chemical composition, physicochemical properties (size, geometry, surface charge, hydrophobicity, biocompatibility, stability in biological fluids), and photoabsorbing properties (optical properties, strong NIR absorption, large extinction coefficient, excellent photostability, good thermal conductivity, and effective generation of acoustic waves). They must also be biocompatible for systemic administration and cross the blood–brain barrier. However, by designing systems that can be directly injected into the tumor or the tumor resection cavity, some of these design constraints can be reduced, whilst allowing appropriate amounts of the photothermal agent to be placed directly at the required site of therapeutic intervention.

Pre-clinical studies have shown that the local delivery of therapeutics can outperform current chemotherapeutic options [7,41,42]. In addition, a recent systematic review and meta-analysis of pre-clinical data showed that local delivery of temozolomide achieved better therapeutic efficacy than systemic administration [43]. These data therefore provide a rationale for the development of such hydrogel delivery systems, despite the limited benefits associated with Gliadel wafers [44,45].

We are aware that this study only reports cytotoxic efficacy in cellular studies, which do not represent the clinical setting. We also acknowledge that without assessing the amount of doxorubicin loaded into the hydrogels, a detailed study of the release kinetics could not be undertaken. However, we hope in the future to be able to address this and demonstrate the potential of this combined system in a pre-clinical setting. Despite this limitation, we believe that the data reported in this paper show a promising future for these innovative hydrogels, which could be loaded with a wide range of drugs and photoabsorbers for an effective combinatory strategy against Glioblastoma.

## 4. Conclusions

In summary, we have developed a polyethylene glycol diacrylate hydrogel drug delivery system that was well defined in terms of dimensions, chemical composition, and drug release profile. A microscale template-assisted synthesis strategy and a high solid content resulted in hydrogels that were robust for handling and reproducible and controlled the release of doxorubicin for 7 days without a substantial initial burst release. Furthermore, PEGylated gold nanorods could be incorporated for additional photothermal therapy. We showed that the hydrogels themselves did not affect C6 glioma cell viability, but when coupled with near-infrared light and/or doxorubicin release, a significant reduction in glioma viability could be achieved. Although no combinatorial effect was observed, this study proves the concept that both photothermal ablation and chemotherapeutic delivery can be achieved from a single hydrogel system. Whilst further research is required to determine the pre-clinical efficacy, this study shows that PEG-based hydrogels offer an alternative biomaterial for local drug delivery strategies.

## Data Availability

All the data reported in this study is shown in this manuscript and in Supplementary Materials.

## Supplementary Materials

The following supporting information can be downloaded at: www.mdpi.com/article/10.3390/pharmaceutics14030551/s1, Figure S1: Analysis of the photothermal properties of 100 µm-width hydrogels loaded with nanorods, when tested on the bench top (at room temperature) whilst still in their templates.
