# Supplementary material for: Well-Defined Polyethylene Glycol Microscale Hydrogel Blocks Containing Gold Nanorods for Dual Photothermal and Chemotherapeutic Therapy
Source: Pharmaceutics. 2022 Feb 28;14(3):551. doi: 10.3390/pharmaceutics14030551 (PMC8954019; doi:10.3390/pharmaceutics14030551)
Supplement: Supplementary file 1 [file pharmaceutics-14-00551-s001.zip › pharmaceutics-1579792-supplementary.pdf]

pharmaceutics

MDPI

Supplementary Materials: Well-Defined Polyethylene Glycol Microscale Hydrogel Blocks Containing Gold Nanorods for Dual Photothermal and Chemotherapeutic Therapy

Ben Newland, Johannes Starke, Chiara Bastiancich, Diana Goncalves-Schmidt, Laura J. Bray, Wenxin Wang and Carsten Werner

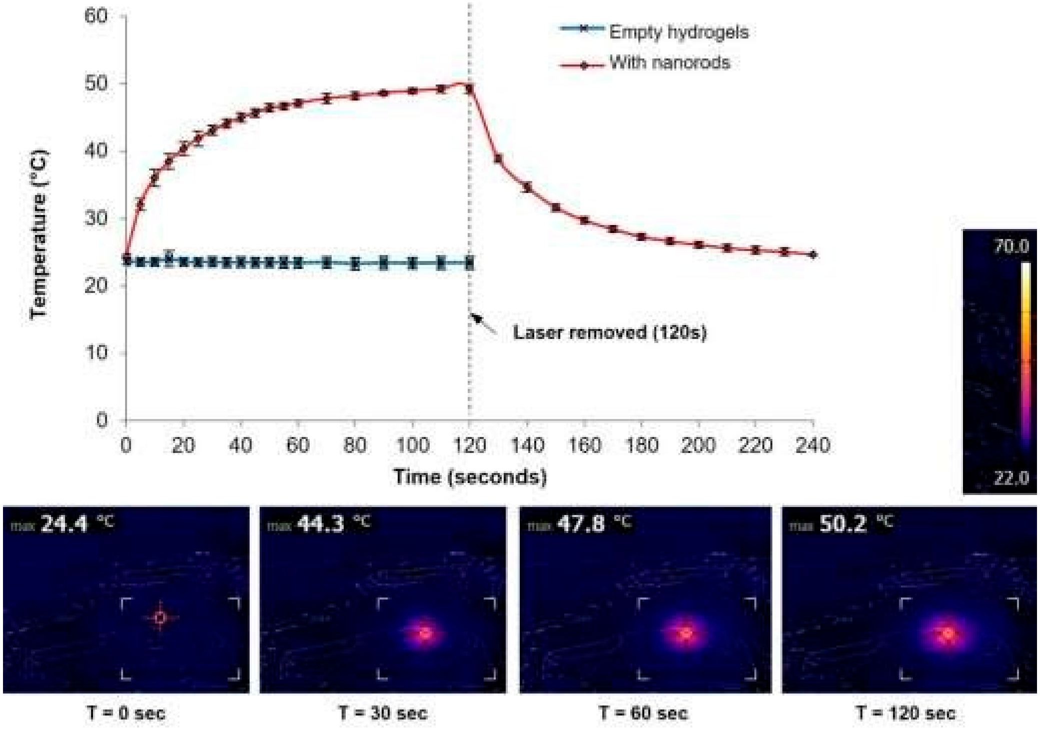

Figure S1. Analysis of the photothermal properties of 100 µm width hydrogels loaded with nanorods, when tested on the bench top (at room temperature) whilst still in their templates. This shows the large increase in temperature which is possible when these hydrogels are in close confinement rather than diluted in suspension in a 96-well plate ($n = 3$, error bars represent ± standard deviation).
